# Erik Satie – a psychopathological approach

**DOI:** 10.1192/j.eurpsy.2021.1821

**Published:** 2021-08-13

**Authors:** M. Gonçalves-Pinho, J.P. Ribeiro

**Affiliations:** Department Of Psychiatry And Mental Health, Centro Hospitalar do Tâmega e Sousa, Penafiel, Portugal, Penafiel, Portugal

**Keywords:** Erik Satie, Music, psychiatry, Personality

## Abstract

**Introduction:**

Éric Satie was a French classical music composer born in May of 1866. He composed several music pieces that did not fit the contemporaneous musical standard once he did not follow the orthodox rules of composition and harmonic expression.

**Objectives:**

To analyse Erik Satie personality traits and possible psychopathological findings.

**Methods:**

A narrative review was performed using Google Scholar database.

**Results:**

His music, as it occurs in most musical composers, was said to translate his own personality and state of mind at the time. He was described as an eccentric with multiple descriptions demonstrating unstable and explosive personality traits of pride, determination, perfectionism and a hatred for convention that would put him near a Cluster A type of personality.

**Conclusions:**

Although some authors conclude that Satie could be diagnosed with Asperger Syndrome I believe that his specificities represent more of personality traits than pathological findings.

**Disclosure:**

No significant relationships.

